# Morphological Type Identification of Self-Incompatibility in *Dendrobium* and Its Phylogenetic Evolution Pattern

**DOI:** 10.3390/ijms19092595

**Published:** 2018-09-01

**Authors:** Shan-Ce Niu, Jie Huang, Qing Xu, Pei-Xing Li, Hai-Jun Yang, Yong-Qiang Zhang, Guo-Qiang Zhang, Li-Jun Chen, Yun-Xia Niu, Yi-Bo Luo, Zhong-Jian Liu

**Affiliations:** 1 Shenzhen Key Laboratory for Orchid Conservation and Utilization, The National Orchid Conservation Center of China and The Orchid Conservation and Research Center of Shenzhen, Shenzhen 518114, China; niushance@163.com (S.-C.N.); 13530401396@163.com (J.H.); xqing1987@163.com (Q.X.); lpxcathy@hotmail.com (P.-X.L.); hjyang@scau.edu.cn (H.-J.Y.); bagoodman@163.com (Y.-Q.Z.); zhang2384539@163.com (G.-Q.Z.); chenlj@sinicaorchid.org (L.-J.C.); 2 State Key Laboratory of Systematic and Evolutionary Botany, Institute of Botany, Chinese Academy of Sciences, Beijing 100093, China; 3 University of Chinese Academy of Sciences, Beijing 100049, China; niuyx@psych.ac.cn; 4 College of Forestry and Landscape Architecture, Center of Experimental Teaching for Common Basic Courses, South China Agricultural University, Guangzhou 510640, China; 5 Key Laboratory of Mental Health, Institute of Psychology, Chinese Academy of Sciences, Beijing 100093, China; 6 College of Forestry and Landscape Architecture, South China Agricultural University, Guangzhou 510640, China; 7 College of Landscape Architecture, Fujian Agriculture and Forestry University, Fuzhou 350002, China; 8 The Center for Biotechnology and BioMedicine, Graduate School at Shenzhen, Tsinghua University, Shenzhen 518055, China

**Keywords:** *Dendrobium*, self-incompatibility, pollen tube, capsule set, ancestral status

## Abstract

Self-incompatibility (SI) is a type of reproductive barrier within plant species and is one of the mechanisms for the formation and maintenance of the high diversity and adaptation of angiosperm species. Approximately 40% of flowering plants are SI species, while only 10% of orchid species are self-incompatible. Intriguingly, as one of the largest genera in Orchidaceae, 72% of *Dendrobium* species are self-incompatible, accounting for nearly half of the reported SI species in orchids, suggesting that SI contributes to the high diversity of orchid species. However, few studies investigating SI in *Dendrobium* have been published. This study aimed to address the following questions: (1) How many SI phenotypes are in Dendrobium, and what are they? (2) What is their distribution pattern in the Dendrobium phylogenetic tree? We investigated the flowering time, the capsule set rate, and the pollen tube growth from the representative species of *Dendrobium* after artificial pollination and analysed their distribution in the Asian *Dendrobium* clade phylogenetic tree. The number of SI phenotypes exceeded our expectations. The SI type of *Dendrobium chrysanthum* was the primary type in the *Dendrobium* SI species. We speculate that there are many different SI determinants in *Dendrobium* that have evolved recently and might be specific to *Dendrobium* or Orchidaceae. Overall, this work provides new insights and a comprehensive understanding of *Dendrobium* SI.

## 1. Introduction

The genus *Dendrobium* in the subfamily Epidendroideae, Orchidaceae, which consists of approximately 1450 species, is one of the largest genera in Orchidaceae. Most of the species in this genus are epiphytes with high ornamental, medicinal, and commercial value and are favoured by botanists and plant enthusiasts [1]. As one of the largest Orchidaceae genera, most (72%) of the 61 *Dendrobium* species that are known to be self-pollinated show self-sterility [2]. Nearly half of orchid self-incompatibility (SI) species are from *Dendrobium*. So, the high SI rate in *Dendrobium* species might contribute to their high level of species diversity.

SI is a type of reproductive barrier within species which can promote outcrossing and maintain heterozygosity. SI, with multiple independent origins, exists in approximately 40% of flowering plants, based on the plant species tested [3,4]. SI is closely related to the formation and maintenance of the high diversity and adaptation of angiosperm species [5,6].

The SI response is controlled by a single highly polymorphic locus, the S-locus, in most reported species. There are at least two closely linked genes (the male and female S-determinants) located at the S-locus, and self and non-self modes are discriminated by a specific molecular interaction between these S-determinants [7]. Based on the modes of genetic control of the pollen SI phenotype, SI can be classified as gametophytic SI (GSI) or sporophytic SI (SSI), in which the pollen SI phenotype is controlled by the haploid pollen itself (Papaveraceae, Solanaceae, Rosaceae, and Plantaginaceae) and diploid donor tissues (anther tapetum in *Brassica* of the Brassicaceae) [5], respectively. There are three types of well-studied SI systems with molecular characterization: Firstly, the self-recognition mechanism of SSI in Brassicaceae, especially in *Brassica*. The male S-determinant is the S-locus protein 11 (SP11) or the S-locus cysteine-rich protein (SCR), a small secreted peptide that localizes in the pollen coat [8,9]. The female S-determinant is the S-locus receptor kinase (SRK), a Ser/Thr receptor kinase localized to the plasma membrane of the papilla cells of the stigma [10]. The interaction between these two S-determinants from the same S-haplotype induces the incompatibility response in the stigma, leading to self-pollen rejection [11,12,13] which belongs to the receptor and ligand interaction type. Secondly, there is the self-recognition mechanism of gametophytic self-incompatibility (GSI) in Papaveraceae, mainly in *Papaver rhoeas*. The interaction between the male S-determinant (*P. rhoeas* pollen S, *PrpS*, pollen surface receptor) and the female S-determinant (*P. rhoeas* style S, *PrsS*, small protein secreted by papilla cells of the stigma) from the same S-haplotype induces the SI response and belongs to the novel “receptor”–ligand interaction type [14]. Thirdly, the non-self-recognition mechanism of GSI in Solanaceae, Rosaceae, and Plantaginaceae, is different from the above two types. The male S-determinant is made up of multiple *S-locus F-box* (*SLF*) genes [15], encoding the F-box proteins [16], and the female S-determinant S-RNase is mainly present as a glycoprotein in the style tissue. Each SLF protein interacts specifically with one or more S-RNase proteins of other S-haplotypes. The set of SLF genes encoded by a single S-haplotype allows pollen to recognize and detoxify multiple allelic variants of non-self S-RNase [15,17], which is called the collaborative non-self-recognition model.

In addition to the differences between the molecular mechanisms among these three SI systems, the pollen tube morphologies of SI also show significant differences. In Solanaceae, the growth of the incompatible pollen tube is arrested by the time it reaches approximately one-third of the way through the style [18]. The rejection of pollen in *Papaver rhoeas* occurs on the stigmatic surface. Inhibition of incompatible pollen in *P. rhoeas* is rapid compared with the relatively slow inhibition in Solanaceae, acting within several minutes [18]. These differences have led to suggestions that GSI systems have evolved independently several times. In *Brassica*, all self-pollen grains fail to adhere strongly to the papilla [19,20], and hydration is arrested before the pollen becomes fully turgid [21]. Different cessation positions of pollen tube growth suggest that they are controlled by different molecular mechanisms. In addition to these three types of SI pollen tube morphology, there is also ovarian SI, in which pollen is rejected at the ovarian interface and its molecular mechanism is unknown [22].

Seed and fruit setting, the growth of pollen tubes, seed filling, seed germination, and seedling development are influenced by SI in Orchidaceae. Therefore, the capsule set, seed filling, and growth of pollen tubes following self- and cross-pollination are the primary SI indicators [23]. Due to the long time between pollination and seed maturation—mostly between 6 months and 1 year—the growth of pollen tubes and the capsule set rate are the most important and feasible markers of the SI response. Studies performed on *Pleurothallidinae* and *Dendrobium* [2,23,24,25] suggest that the sites of incompatibility reactions vary among different groups of orchids, implying that different SI molecular mechanisms exist in Orchidaceae. In some species, such as *Masdevallia infracta*, *Octomeria*, *Stelis*, *Specklinia*, and *Anathallis* spp. (except *A. microphyta*), pollen after pollination does not germinate [26], and self-pollination of 26 species of *Restrepia* (Orchidaceae) revealed that pollen tubes grow only into the top third of the ovary [23], while *Pleurothallis adamantinensis* and *P. fabiobarrosii* are strictly self-incompatible because pollen tube growth ceases near the base of the style [27]. We identified two SI species in *Dendrobium*: *Dendrobium longicornu* and *D. chrysanthum*. The growth of the self-pollinated pollen tube in both species ceases at the top of the style [28].

Even studies have shown a high SI species rate in *Dendrobium*, the morphology of pollen tube growth, its distribution pattern, and its ancestral status in the *Dendrobium* phylogenetic tree remain unclear. As previously mentioned, we have already identified two SI species (*D. longicornu* and *D. chrysanthum*) by investigating the growth of pollen tubes using fluorescence microscopy [28]. In this study, in order to know the potential pollen tube morphology in *Dendrobium*, we carried out a systematic investigation on the capsule set rate and pollen tube growth over 2 years. Thereafter, we analysed the distribution pattern of the capsule set rate after pollination in the *Dendrobium* phylogenetic tree, the distribution pattern of pollen tube growth of SI species in the *Dendrobium* phylogenetic tree, and its ancestral status. We conclude that there are several different sites at which the growth of the pollen tube bundle of self-pollinated species stops and the diverse pollen tube bundle phenotypes in *Dendrobium* SI species, suggesting that different SI molecular bases might exist between SI species with different pollen morphologies, and that the SI morphologies of the growth of the pollen tube bundle that stopped at the upper one-third of the style might be the main SI type.

## 2. Results

### 2.1. The Results Obtained from Artificial Pollination

Self- and cross-pollination were performed in 26 *Dendrobium* species over a 2-year period (Table 1). Flowering time for a single flower is generally longer than 6 days, and the longest is 21 days, such as in *D. cariniferum*. Flowering times of most species are between 8 and 11 days, such as in *D. catenatum*, *D. chrysanthum*, and *D. longicornu*, while those of *D. farmer* and *D. stuposum* are the shortest, only 6 days.

In regard to the capsule set rate (CSR), there were some self-sterile species, such as *D. aphyllum*, *D. densiflorum*, *D. devonianum*, *D. farmer*, *D. hancockii*, *D. jenkinsii*, *D. longicornu*, *D. moniliforme*, *D. polyanthum*, and *D. stuposum*. Of these species, we also found a CSR with cross-pollination of zero in *D. devonianum* and *D. longicornu*. However, the CSR was 100% in *D. longicornu* planted in Malipo, Yunnan Province, China. Our hypothesis is that these two species might not be adapted to the environments of Shenzhen, even after being planted for more than 8 years. In addition, the cross-pollinated capsule set rate (CPCSR) for most species is higher than 50% and reaches nearly 100%, except for *D. moniliforme* and *D. stuposum*, which are 34–41% and 37.5–50%, respectively.

We also found some species with a low self-pollinated capsule set rate (SPCSR) (1–24%). These are *D. aduncum*, *D. cariniferum*, *D. catenatum*, *D. chrysanthum*, *D. fimbriatum*, *D. gratiosissimum*, *D. harveyanum*, *D. lindleyi*, *D. thyrsiflorum*, and *D. unicum*. In these species, the CPCSRs of *D. aduncum*, *D. cariniferum*, *D. catenatum*, and *D. unicum* are between 0% and 60%. The CSR of *D. unicum* showed very significant differences between the two years—the CPCSR was 60% in 2015 and 0% in 2016, while the SPCSR was 0% in 2015 and 21.4% in 2016.

Six species, *D. chrysotoxum*, *D. crystallinum*, *D. denneanum*, *D. hainanense*, *D. hercoglossum*, and *D. linawianum,* had a relatively high CSR—35–100% for self-pollination and 41–100% for cross-pollination. *Dendrobium hercoglossum* also showed large differences in CSR values between the 2 years, with 0% and 57% for self-pollination in 2015 and 2016, respectively, and 36% and 15.5% for cross-pollination in 2015 and 2016, respectively. These differences might also be caused by environmental influences.

### 2.2. Pollen Tube Growth Results

Based on the results from artificial pollination and the phylogenetic tree of *Dendrobium*, we selected the species containing all types of CSR and phylogenetic clades and observed pollen tube growth using fluorescence microscopy after self- and cross-pollination over 2 years.

**Figure 1:**
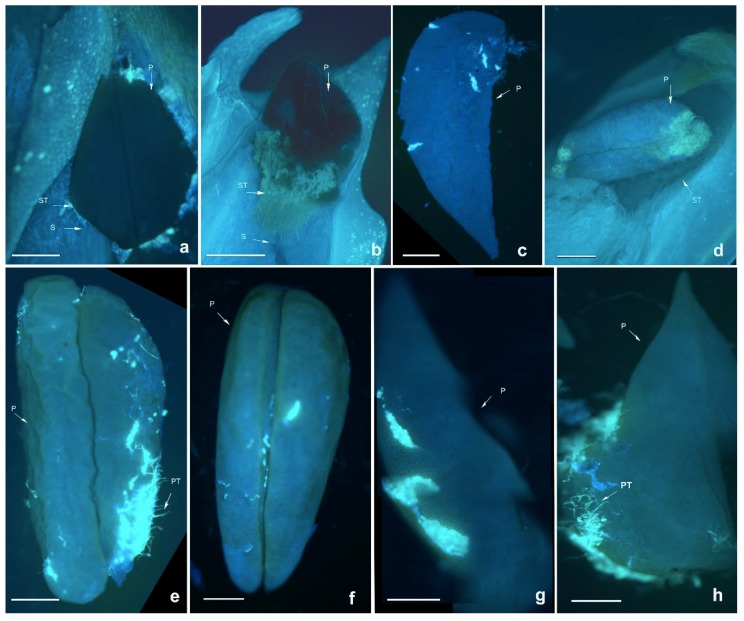
Growth of pollen tubes after self- and cross-pollination of *Dendrobium longicornu* in Shenzhen. PT: pollen tube; P: pollinium; S: style; ST: stigma. (**a**) 2 h after cross-pollination; (**b**) 24 h after cross-pollination; (**c**) 48 h after cross-pollination; (**d**) 96 h after cross-pollination; (**e**) 2 h after self-pollination; (**f**) 24 h after self-pollination; (**g**) 48 h after self-pollination; (**h**) 96 h after self-pollination. Bars = 300 µm.

A previous report of *D. chrysanthum* and *D. longicornu* (Malipo, Yunnan) [28] stated that *D. longicornu* planted in Shenzhen had no seeds after self- and cross-pollination. The pollen tube grew very slowly and was short, with a membrane-like substance coating the pollinium (Figure 1); which might inhibit the germination of the pollen tube and hinder the pollen tube growth, providing resistance against the unadaptable environment.

**Figure 2:**
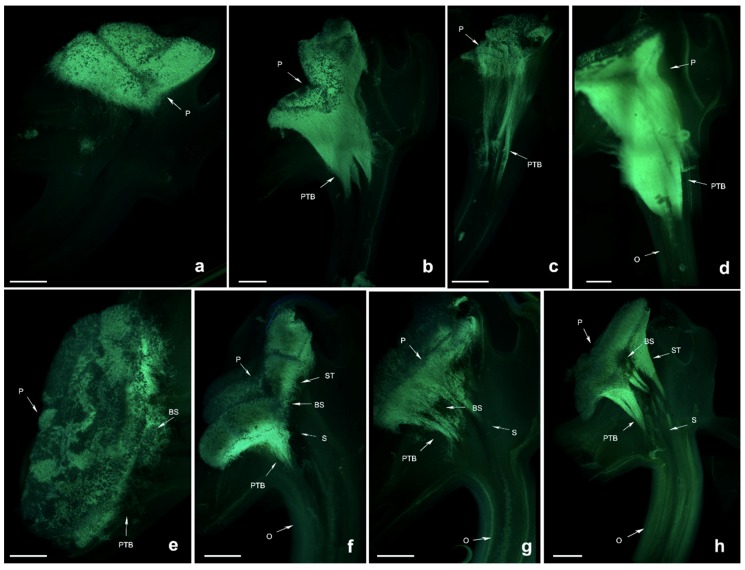
Growth of pollen tubes after self- and cross-pollination of *Dendrobium lindleyi*. P: pollinium; PTB: pollen tube bundle; BS: black spots; O: ovary; ST: stigma; S: style. (**a**) 24 h after cross-pollination; (**b**) 36 h after cross-pollination; (**c**) 48 h after cross-pollination; (**d**) 72 h after cross-pollination; (**e**) 24 h after self-pollination; (**f**) 36 h after self-pollination; (**g**) 48 h after self-pollination; (**h**) 72 h after self-pollination. Bars = 300 µm.

*Dendrobium lindleyi* is a self-sterile species based on artificial pollination (Table 1). Compared with the pollen tube bundle (PTB) of cross-pollinated plants, the PTB of self-pollinated plants grew more slowly up to 48 h after self-pollination (HASP), when the PTB was at the bottom of the style or had just reached the ovary. Next, the PTB stopped growing. Finally, flower abscission happened. At 24 HASP, black spots began to appear which increased in number over time. This might be due to the degradation of callose in the pollen tube which cannot make the pollen tube fluorescent, leading to the SI phenotype. However, the PTB of cross-pollinated species grew much faster, reaching the ovaries in 36 or 48 HASP (Figure 2).

**Figure 3:**
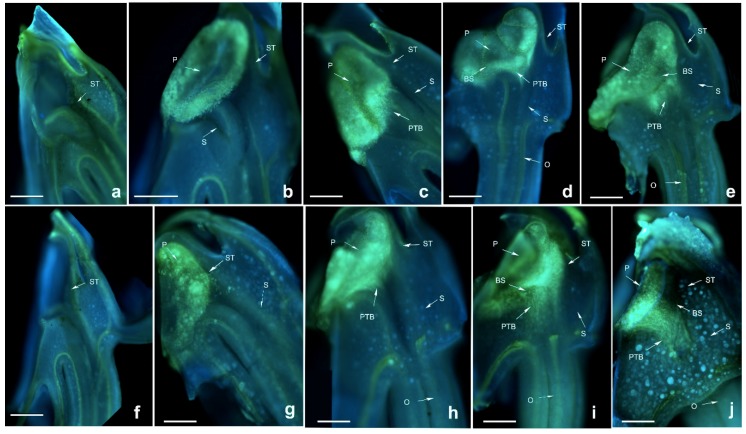
Growth of pollen tubes after self- and cross-pollination of *Dendrobium devonianum.* ST: stigma; P: pollinium; S: style. BS: black spots; PTB: pollen tube bundle; O: ovary. (**a**) 24 h after cross-pollination; (**b**) 48 h after cross-pollination; (**c**) 72 h after cross-pollination; (**d**) 96 h after cross-pollination; (**e**) 120 h after cross-pollination; (**f**) 24 h after self-pollination; (**g**) 48 h after self-pollination; (**h**) 72 h after self-pollination; (**i**) 96 h after self-pollination; (**j**) 120 h after self-pollination. Bars = 100 µm.

Pollen tubes of *D. devonianum* showed similar growth rates after self- and cross-pollination, and black spots appeared in the pollinium and pollen tubes at both 96 HASP and HACP (hours after cross pollination), when the PTB grew up to the upper end of the style (Figure 3). The zone of black spots became larger at 120 HASP and HACP, indicating the degradation of callose, meaning that the pollen tube cannot be stained. This might be a different phenotype from that of *D. longicornu*, showing the lack of adaptability to the environment. These results imply that a style substance or release of some materials from the style already exists when the pollen tube grows up to a certain position in the style that degrades the pollen tube, which shows the self- and cross-incompatibility phenotype.

**Figure 4:**
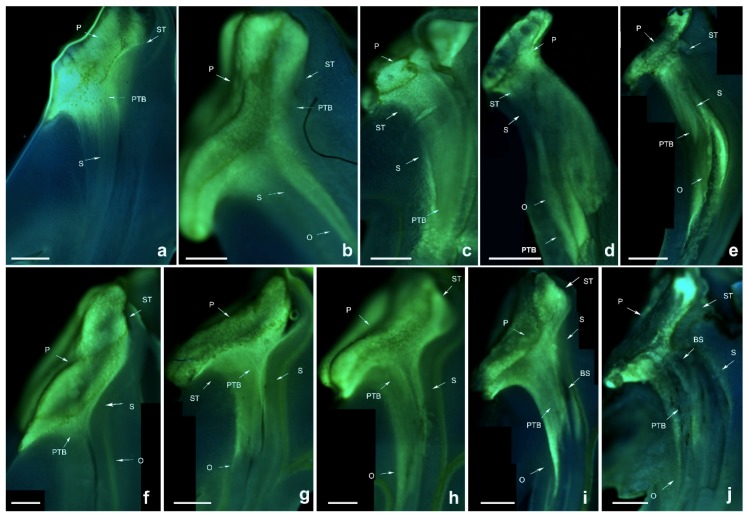
Growth of pollen tubes after self- and cross-pollination of *Dendrobium denneanum.* P: pollinium; ST: stigma. BS: black spots; PTB: pollen tube bundle; S: style; O: ovary. (**a**) 24 h after cross-pollination; (**b**) 48 h after cross-pollination; (**c**) 72 h after cross-pollination; (**d**) 96 h after cross-pollination; (**e**) 120 h after cross-pollination; (**f**) 24 h after self-pollination; (**g**) 48 h after self-pollination; (**h**) 72 h after self-pollination; (**i**) 96 h after self-pollination; (**j**) 120 h after self-pollination. Bars = 100 µm.

*Dendrobium denneanum* is a self- and cross-fertilised species that is present in most individuals based on the artificial pollination results. During pollen tube growth of self-sterile individuals, black spots were present after 96 h of self-pollination, and the zone of black spots became larger until the pollen tube grew into the ovary (Figure 4). This is similar to *D. devonianum*, except that the growth of the pollen tube in *D. devonianum* stops at the upper end of the style. This is also similar to pollen tube growth in *D. lindleyi*, but the black spots appear later than in *D. lindleyi*.

**Figure 5:**
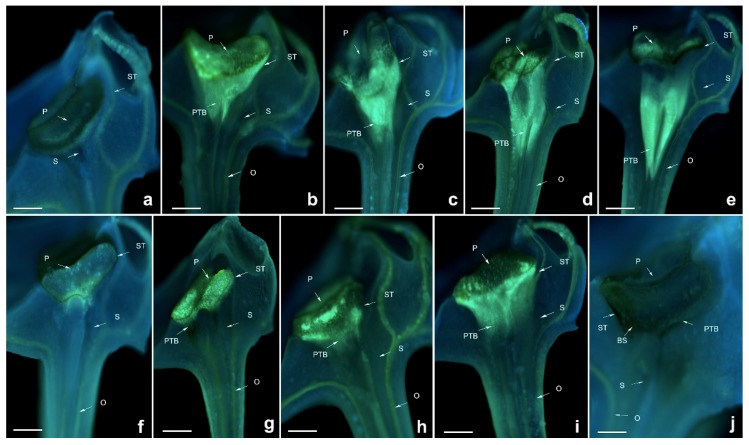
Growth of pollen tubes after self- and cross-pollination of *Dendrobium unicum*. P: pollinium; ST: stigma; S: style; BS: black spots; PTB: pollen tube bundle; O: ovary. (**a**) 24 h after cross-pollination; (**b**) 48 h after cross-pollination; (**c**) 72 h after cross-pollination; (**d**) 96 h after cross-pollination; (**e**) 120 h after cross-pollination; (**f**) 24 h after self-pollination; (**g**) 48 h after self-pollination; (**h**) 72 h after self-pollination; (**i**) 96 h after self-pollination; (**j**) 120 h after self-pollination. Bars = 100 µm.

Because of the significantly different CSR results in *D. unicum* between the 2 years, we analyse the pollen tube growth of self-sterile plants. The growth of PTB in *D. unicum* was similar to that in *D. devonianum*, *D. denneanum*, and *D. lindleyi*, and all of them had black spots (Figure 5). However, the growth cessation position of PTB was at a certain position of the style in *D. unicum* and *D. devonianum*, while it was at the ovary and the bottom of the style or the upper part of the ovary in *D. denneanum* and *D. lindleyi*, respectively*.*

**Figure 6:**
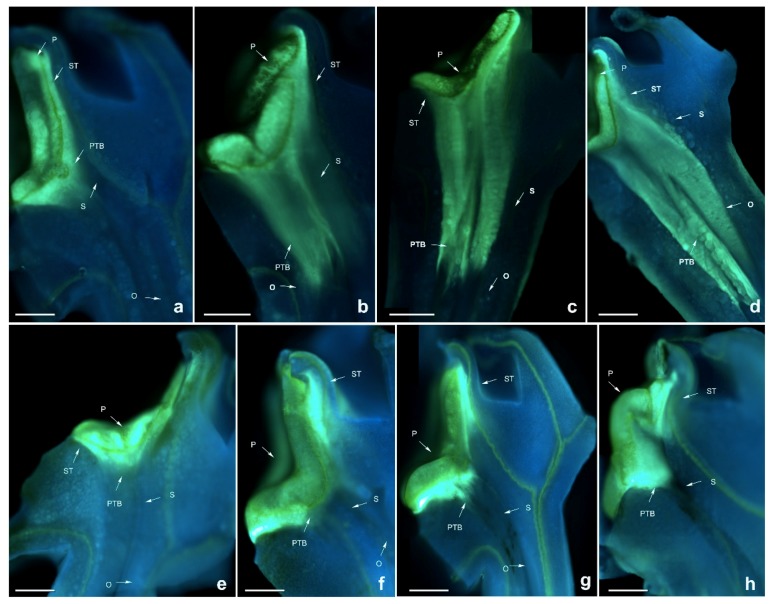
Growth of pollen tubes after self- and cross-pollination of *Dendrobium densiflorum*. P: pollinium; ST: stigma; PTB: pollen tube bundle; S: style; O: ovary. (**a**) 24 h after cross-pollination; (**b**) 48 h after cross-pollination; (**c**) 72 h after cross-pollination; (**d**) 96 h after cross-pollination; (**e**) 24 h after self-pollination; (**f**) 48 h after self-pollination; (**g**) 72 h after self-pollination; (**h**) 96 h after self-pollination. Bars = 100 µm.

Based on the artificial pollination results, *Dendrobium densiflorum* is a self-sterile species. The pollen tube grew fast after cross-pollination, especially at 48 HACP when the PTB had grown into the ovary (Figure 6). Although the self-pollination PTB grew much more slowly, there was no growth 48 h after self-pollination, and the growth of PTB stopped at the surface of the stigma or when just entering the style (Figure 6). We speculate that some substance from the style blocked the growth of the pollen tube, or the blocking response occurred earlier on and the phenotype appearance was delayed.

*Dendrobium catenatum* has partial self- and partial cross-sterile characteristics. The SPCSR was 11% and 10% in 2015 and 2016, while the CPCSR was 23% and 40% in 2015 and 2016, respectively (Table 1). For self-incompatible individuals, PTB growth stops at the upper end of the style 48 or 72 h after self-pollination. At nearly the same time, black spots appear in the pollinium and the pollen tube and increase in number over time. This phenotype is also similar to those of *D. unicum*, *D. denneanum*, *D. lindleyi*, and *D. devonianum*, although pollen tube growth positions and the timing of the appearance of black spots are different.

**Figure 7:**
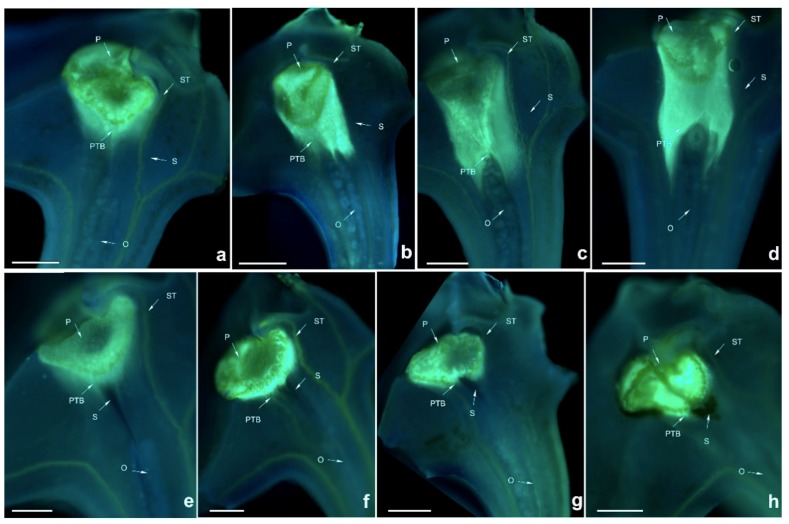
Growth of pollen tubes after self- and cross-pollination of *Dendrobium moniliforme*. P: pollinium; ST: stigma; PTB: pollen tube bundle; S: style; O: ovary. (**a**) 48 h after cross-pollination; (**b**) 72 h after cross-pollination; (**c**) 96 h after cross-pollination; (**d**) 120 h after cross-pollination; (**e**) 48 h after self-pollination; (**f**) 72 h after self-pollination; (**g**) 96 h after self-pollination; (**h**) 120 h after self-pollination. Bars = 100 µm.

*Dendrobium moniliforme* is a self-sterile and partial cross-fertile species. The SPCSR was 0% over the 2 years, and the CPCSR was 34% and 41% in 2015 and 2016, respectively (Table 1). We chose the self-sterile and cross-fertile plants by observing the growth of the pollen tube. The PTB values between self- and cross-pollination were significantly different 72 h after pollination, and PTB growth stopped at the upper end of the style (Figure 7).

**Figure 8:**
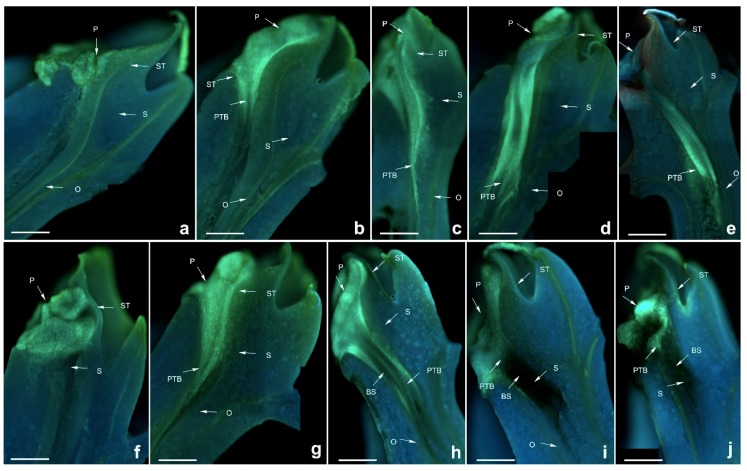
Growth of pollen tubes after self- and cross-pollination of *Dendrobium hancockii*. P: pollinium; ST: stigma; S: style; O: ovary; BS: black spots; PTB: pollen tube bundle. (**a**) 24 h after cross-pollination; (**b**) 48 h after cross-pollination; (**c**) 72 h after cross-pollination; (**d**) 96 h after cross-pollination; (**e**) 120 h after cross-pollination; (**f**) 24 h after self-pollination; (**g**) 48 h after self-pollination; (**h**) 72 h after self-pollination; (**i**) 96 h after self-pollination; (**j**) 120 h after self-pollination. Bars = 100 µm.

**Figure 9:**
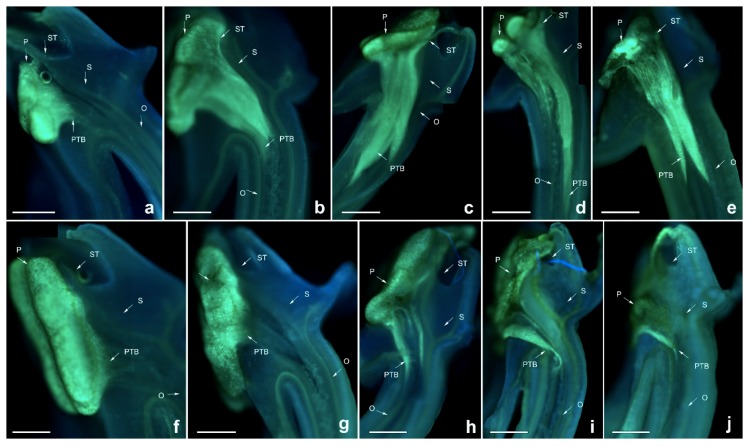
Growth of pollen tubes after self- and cross-pollination of *Dendrobium jenkinsii*. P: pollinium; ST: stigma; S: style; PTB: pollen tube bundle; O: ovary. (**a**) 24 h after cross-pollination; (**b**) 48 h after cross-pollination; (**c**) 72 h after cross-pollination; (**d**) 96 h after cross-pollination; (**e**) 120 h after cross-pollination; (**f**) 24 h after self-pollination; (**g**) 48 h after self-pollination; (**h**) 72 h after self-pollination; (**i**) 96 h after self-pollination; (**j**) 120 h after self-pollination. Bars = 100 µm.

*Dendrobium hancockii* is an artificially pollinated self-sterile species with 0% SPCSR and 100% CPCSR (Table 1). There was no difference in PTB growth between self- and cross-pollination, and the PTB of self-pollinated plants also grew into the ovaries. Surprisingly, black spots also appeared 72 h after self-pollination, and the black zone increased in size until no pollen tube was observed 120 h after self-pollination (Figure 8). Pollen tube growth in *D. jenkinsii* after self- and cross-pollination was similar to that of *D. hancockii* and *D. lindleyi* (Figure 9), suggesting that they might have the same SI molecular mechanism.

Based on the results of self- and cross-pollination and pollen tube growth, we observed a high diversity of self- and cross-sterility and thus many different types of self-incompatible pollen tube morphologies. Therefore, what is the distribution pattern and what is the ancestral status of this morphology?

### 2.3. Distribution Pattern and Ancestral Status of Self-Incompatibility and Self-Compatibility in Asian Dendrobium Species

We combined the CSR and pollen tube growth results after pollination and identified self-incompatible species and self-compatible species. using the reported *Dendrobium* self-incompatible species [2] and species in this study, we analysed the self-incompatibility and self-compatibility distribution pattern and ancestral status of the mating system in *Dendrobium* from Asia.

There were 61 species used in this analysis. Twenty-six species were from this study, and the others were from unpublished data and data from Johansen [2]. As we were limited by the materials, we focused on the Asian clade of *Dendrobium*.

**Figure 10:**
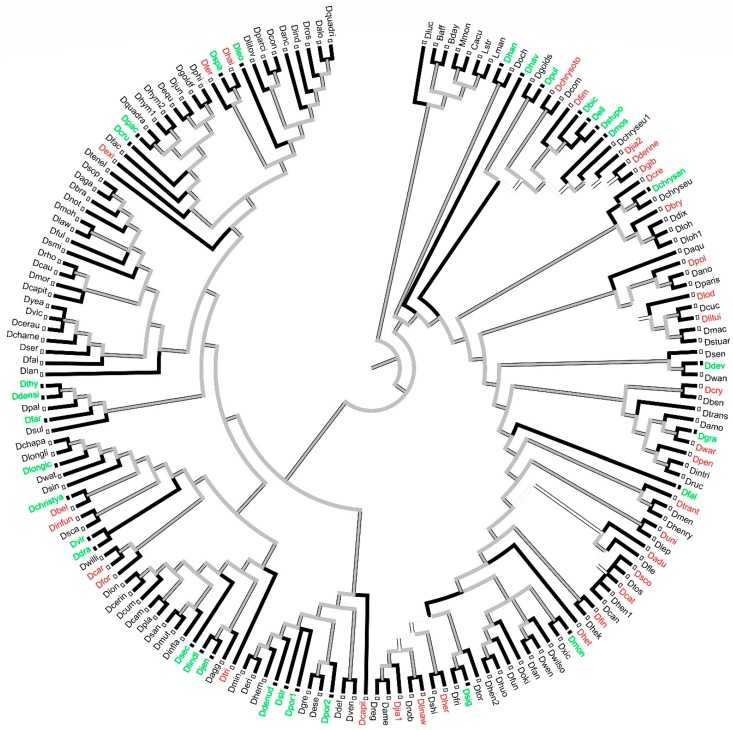
Parsimony inference of the mating system evolution of *Dendrobium.* Red words: self-compatibility; green words: self-incompatibility. Lman, Bday, Baff, Lstr, Cacu, Mmon, and Dluc were used as outgroups. Baff: *Bulbophyllum affine*; Bday: *Bulbophyllum dayanum*; Cacu: *Crepidium acuminatum*; Dadu: *Dendrobium aduncum*; Daga: *Dendrobium agathodaemonis*; Dagg: *Dendrobium aggregatum*; Dalo: *Dendrobium aloifolium*; Dame: *Dendrobium amethystoglossum*; Damo: *Dendrobium amoenum*; Danc: *Dendrobium anceps*; Dano: *Dendrobium anosmum*; Daqu: *Dendrobium aqueum*; Dbel: *Dendrobium bellatulum*; Dben: *Dendrobium bensoniae*; Dbic: *Dendrobium bicameratum*; Dbra: *Dendrobium bracteosum*; Dbry: *Dendrobium brymerianum*; Dcam: *Dendrobium camptocentrum*; Dcan: *Dendrobium candidum*; Dcapil: *Dendrobium capillipes*; Dcapit: *Dendrobium capituliflorum*; Dcar: *Dendrobium cariniferum*; Dcat: *Dendrobium catenatum*; Dcau: *Dendrobium cauliculimentum*; Dcerin: *Dendrobium cerinum*; Dcerau: *Dendrobium ceraula*; Dchapa: *Dendrobium chapaense*; Dchame: *Dendrobium chameleon*; Dchrysan: *Dendrobium chrysanthum*; Dchryseu: *Dendrobium chryseum*; Dchrysoto: *Dendrobium chrysotoxum*; Dchristya: *Dendrobium christyanum*; Dcom: *Dendrobium compactum*; Dcon: *Dendrobium confusum*; Dcre: *Dendrobium crepidatum*; Dcru: *Dendrobium crumenatum*; Dcry: *Dendrobium crystallinum*; Dcuc: *Dendrobium cucullatum*; Dcum: *Dendrobium cumulatum*; Ddel: *Dendrobium delacourii*; Ddenne: *Dendrobium denneanum*; Ddenud: *Dendrobium denudans*; Ddensi: *Dendrobium densiflorum*; Ddev: *Dendrobium devonianum*; Ddix: *Dendrobium dixanthum*; Ddra: *Dendrobium draconis*; Dell: *Dendrobium ellipsophyllum*; Dequ: *Dendrobium equitans*; Deri: *Dendrobium eriiflorum*; Dese: *Dendrobium eserre*; Dexi: *Dendrobium exile*; Dfac: *Dendrobium faciferum*; Dfai: *Dendrobium fairchildae*; Dfal: *Dendrobium falconeri*; Dfan: *Dendrobium fanjingshanense*; Dfar: *Dendrobium farmer*; Dfim: *Dendrobium fimbriatum*; Dfin: *Dendrobium findlayanum*; Dfle: *Dendrobium flexicaule*; Dfor: *Dendrobium formosum*; Dfri: *Dendrobium friedricksianum*; Dful: *Dendrobium fulgidum*; Dfun: *Dendrobium funiushanense*; Dgib: *Dendrobium gibsonii*; Dgoldf: *Dendrobium goldfinchii*; Dgolds: *Dendrobium goldschmidtianum*; Dgra: *Dendrobium gratiosissimum*; Dgre: *Dendrobium gregulus*; Dhai: *Dendrobium hainanense*; Dhan: *Dendrobium hancockii*; Dhav: *Dendrobium harveyanum*; Dhek: *Dendrobium hekouense*; Dhem: *Dendrobium hemimelanoglossum*; Dhen1: *Dendrobium henanense*; Dhen2: *Dendrobium henanense*; Dhenry: *Dendrobium henryi*; Dher: *Dendrobium hercoglossum*; Dhet: *Dendrobium heterocarpum*; Dhoo: *Dendrobium hookerianum*; Dhuo: *Dendrobium huoshanense*; Dhym1: *Dendrobium hymenanthum*; Dhym2: *Dendrobium hymenanthum*; Dind: *Dendrobium indivisum*; Dinfun: *Dendrobium infundibulum*; Dinfla: *Dendrobium inflatum*; Dion: *Dendrobium ionopus*; Djen: *Dendrobium jenkinsii*; Djia1: *Dendrobium jiaolingense*; Djia2: *Dendrobium jiaolingense*; Djun: *Dendrobium junceum*; Dlan: *Dendrobium lancifolium*; Dlaw: *Dendrobium lawesii*; Dleo: *Dendrobium leonis*; Dlep: *Dendrobium leptocladum*; Dlinaw: *Dendrobium linawianum*; Dlindl: *Dendrobium lindleyi*; Dlitui: *Dendrobium lituiflorum*; Dlitov: *Dendrobium litoveum*; Dlod: *Dendrobium loddigesii*; Dlongic: *Dendrobium longicornu*; Dlongli: *Dendrobium longlingense*; Dloh: *Dendrobium lohohense*; Dloh1: *Dendrobium lohohense*; Dluc: *Dendrobium lucmerianum*; Dmac: *Dendrobium macrostachyum*; Dmen: *Dendrobium menglaense*; Dmin: *Dendrobium minutiflorum*; Dmoh: *Dendrobium mohlianum*; Dmon: *Dendrobium moniliforme*; Dmor: *Dendrobium morrisonii*; Dmos: *Dendrobium moschatum*; Dmut: *Dendrobium mutabile*; Dnob: *Dendrobium nobile*; Dnot: *Dendrobium nothofagicola*; Doch: *Dendrobium ochreatum*; Doki: *Dendrobium okinawense*; Dpac: *Dendrobium pachyphyllum*; Dpal: *Dendrobium palpebrae*; Dparis: *Dendrobium parishii*; Dparci: *Dendrobium parciflorum*; Dpen: *Dendrobium pendulum*; Dphi: *Dendrobium philippinense*; Dpla: *Dendrobium platygastrium*; Dpol: *Dendrobium polyanthum;* Dpor1: *Dendrobium porphyrochilum*; Dpor2: *Dendrobium porphyrochilum*; Dintri: *Dendrobium pseudointricatum*; Dtenel: *Dendrobium pseudotenellum*; Dpul: *Dendrobium pulchellum*; Dquadra: *Dendrobium quadrangulare*; Dquadri: *Dendrobium quadrilobatum*; Dreg: *Dendrobium regium*; Drho: *Dendrobium rhododioides*; Dros: *Dendrobium rosellum*; Druc: *Dendrobium ruckeri*; Dsan: *Dendrobium sanguinolentum*; Dsca: *Dendrobium scabrilingue*; Dsco: *Dendrobium scoriarum*; Dsec: *Dendrobium secundum*; Dsen: *Dendrobium senile*; Dser: *Dendrobium serratilabium*; Dshi: *Dendrobium shixingense*; Dsig: *Dendrobium signatum*; Dsin: *Dendrobium sinense*; Dsmi: *Dendrobium smillieae*; Dsop: *Dendrobium sophronites*; Dspa: *Dendrobium spatella*; Dstr: *Dendrobium strongylanthum*; Dstuar: *Dendrobium stuartii*; Dstupo: *Dendrobium stuposum*; Dsul: *Dendrobium sulcatum*; Dter: *Dendrobium terminale*; Dthy: *Dendrobium thyrsiflorum*; Dtor: *Dendrobium tortile*; Dtos: *Dendrobium tosaense*; Dtrant: *Dendrobium trantuanii*; Dtrans: *Dendrobium transparens*; Dtri: *Dendrobium trigonopus*; Duni: *Dendrobium unicum*; Dven: *Dendrobium venustum*; Dvic: *Dendrobium victoriae-reginae*; Dvir: *Dendrobium virgineum*; Dwan: *Dendrobium wangliangii*; Dwar: *Dendrobium wardianum*; Dwat: *Dendrobium watti*; Dwen: *Dendrobium wenshanense*; Dwilso: *Dendrobium wilsonii*; Dwilli: *Dendrobium williamsonii*; Dxic: *Dendrobium xichouense*; Dyea: *Dendrobium yeageri*; Lman: *Liparis mannii*; Lstr: *Liparis stricklandiana*; Mmon: *Malaxis monophyllos*.

From the distribution of selected species, the samples represented all of the Asian clade (Figure 10). The selected self-compatibility species were located from the top to the bottom of the Asian *Dendrobium* clade, in the order of *D. hainanense*, *D. terminale*, *D. exile*, *D. bellatulum*, *D. infundibulum*, *D cariniferum*, *D. formosum*, *D. trigonopus*, *D. capillipes*, *D.jiaolingense*, *D. linawianum*, *D. hercoglossum*, *D. heterocarpum*, *D. findlayanum*, *D. scoriarum*, *D. catenatum*, *D. aduncum*, *D. unicum*, *D. trantuanii*, *D. wardianum*, *D. pendulum*, *D. crystallinum*, *D. lituiflorum*, *D. loddigesii*, *D. polyanthum*, *D. brymerianum*, *D. crepidatum*, *D. denneanum*, *D. gibsonii*, *D. fimbriatum*, *and D. chrysotoxum*. For the self-incompatibility species, their distribution was the same as that of self-compatibility species. From the top to the bottom of the Asian *Dendrobium* clade, they were *D. leonis*, *D. spatella*, *D. pachyphyllum*, *D. crumenatum*, *D. densiflorum*, *D. thyrsiflorum*, *D. farmer*, *D. longicornu*, *D. christyanum*, *D. draconis*, *D. virgineum*, *D. secundum*, *D. jenkinsii*, *D. lindleyi*, *D. denudans*, *D. porphyrochilum*, *D. strongylanthum*, *D. signatum*, *D. moniliforme*, *D. falconeri*, *D. gratiosissimum*, *D. devonianum*, *D. chrysanthum*, *D. moschatum*, *D. ellipsophyllum*, *D. stuposum*, *D. bicameratum*, *D. pulchellum*, *D. harveyanum*, *and D. hancockii*. Moreover, nearly each subclade had self-incompatibility and self-compatibility species at the same time.

There were four different self-incompatible phenotypes of pollen tube growth as follows: (1) Membrane-like body coating the pollinium hindered pollinium germination and pollen tube growth, such as in *D. longicornu* (Shenzhen); (2) pollen tube growth stopped at the upper end of the style, such as in *D. chrysanthum*, *D. moniliforme*, *D. longicornu* (Malipo), and *D. densiflorum*; (3) no difference in pollen tube growth between self- and cross-pollinated plants, but black spots appeared on the pollen tube when it grew to the bottom of the style or just into the ovary, causing degradation in the self-pollinated pollen tube, for example, in *D. hancockii*, *D. jenkinsii*, and *D. lindleyi*; (4) pollen tube growth stopped at a certain position of the style and black spots appeared in *D. devonianum* and *D. unicum*. The second SI type had a wider phylogenetic distribution, covering the bottom and top locations of the phylogenetic tree, and might be the main SI phenotype in *Dendrobium*.

Based on the ancestral status reconstruction results, self-compatibility was the ancestral status in most subclades, but this was equivocal in the *Dendrobium* clade.

## 3. Discussion

### 3.1. Artificial Pollination and CSR

In this study, 26 species were investigated over 2 years. For certain species with a limited number of individuals, there were comparatively less pollinated flowers, only eight to ten flowers, so more pollination may have been required to reveal the real situation. Nevertheless, we carried out the pollination experiments over 2 years to make up for the lack of quantity. For most species with many individuals, there were sufficient pollinated flowers, i.e., more than 100 flowers. We converted the results from more than 100 flowers to 100 flowers for normalization.

We identified nine species with complete self-sterility in this study. These were *D. aphyllum*, *D. densiflorum*, *D. devonianum*, *D. farmer*, *D. hancockii*, *D. jenkinsii*, *D. longicornu* (Malipo), *D. moniliforme*, and *D. stuposum*, including the cross-sterility species *D. devonianum* and the partial cross-sterility species *D. moniliforme*, *D. aphyllum*, *D. stuposum*, and *D. jenkinsii*.

There were species with different results between the 2 years, such as *D. unicum*, for which the SPCSR was 0% in 2015 and 21.4% in 2016, while the number of pollinated flowers in 2016 was less than that in 2015, at 52 in 2015 and 28 in 2016. This difference might have been caused by the different levels of pollination of plants between the 2 years or changes in the environment. Because of the variation in self-sterility, we treated the species as partially self-incompatible in the following analysis.

One species was also reported by Johansen [2]. The *D. lindleyi* results reported by Johansen showed self- and cross-incompatibility. However, the results from our study showed self-incompatible and cross-compatible species, which was different from the results of Johansen. In the study by Johansen, cross-pollination was carried out in only two flowers from one individual, which was unrepresentative. In our study, we carried out a pollination experiment in at least 100 flowers. Therefore, we used our results for the next analysis.

### 3.2. Diversity of Pollen Tube Growth

We analysed nine species containing all the pollen tube growth types from 26 *Dendrobium* species. The high diversity observed, such as the membrane-like body coating of the pollinium that inhibits germination and limits growth in *D. longicornu* (Shenzhen), exceeded our expectations. For *D. devonianum*, the pollen tubes of self- and cross-pollinated species grew into the style and stopped at a certain position; then, black spots appeared on the pollen tube and the pollinium, leading to self- and cross-incompatibility. These spots might be another phenotype that is resistant to adverse situations, in contrast to *D. longicornu*. The third phenotype was found in *D. jenkinsii*, *D. hancockii*, and *D. lindleyi*. The pollen tube of self-pollinated species grew into the ovary or at the bottom of the style, and then black spots appeared, but the appearance time was earlier than that in *D. devonianum*, which is 72 and 96 h, respectively. In addition, pollen tube growth stopped at the upper part of the style after self-pollination in *D. chrysanthum*, *D. densiflorum*, *D. longicornu* (Malipo), and *D. thyrsiflorum*. In this type, the time at which pollen tube growth stopped in *D. longicornu* was much longer than that in the other three species (96 h and 24–48 h, respectively). For the different times of pollen tube growth cessation, we speculate that the SI determinants of *D. longicornu* might be different from that of *D. chrysanthum*, *D. densiflorum*, and *D. thyrsiflorum*.

Pollen tube growth stopped at different positions in the style or ovary, which is consistent with previous reports in other orchids. For example, pollen tube growth stopped at certain style positions in *P. adamantinensis* and *P. fabiobarrosii*, while it stopped at the ovary in *Restrepia* species, and the pollinia did not germinate in *M. infracta*, *Octomeria*, *Stelis*, and *Specklinia* [2,23,26,27]. This suggests that there are a variety of SI determinants in the Orchidaceae, even in *Dendrobium*.

### 3.3. Distribution Pattern of Self-Incompatibility and Self-Compatibility

Next, we investigated how the different degrees of self- and cross-incompatibility and types of pollen tube growth states after self-pollination are distributed in the *Dendrobium* phylogenetic tree as well as investigating their ancestral statuses.

We concluded that the SI type represented by *D. chrysanthum* was distributed from the bottom to the top of the *Dendrobium* phylogenetic tree, which might be the main SI type of *Dendrobium*, while other SI types were distributed in certain phylogenetic positions. Therefore, we consider that SI in *Dendrobium* has undergone many independent evolutionary processes, controlled by multiple SI determinants.

By reconstructing the ancestral status of SI in the Asian *Dendrobium* clade, we found that self-compatibility was the SI ancestral status in most Asian *Dendrobium* subclades, which was consistent with the findings of Xu but inconsistent with those in Cruciferae and Solanaceae [29,30]. As the majority of orchid species are self-compatible, species at the root of the Orchidaceae phylogenetic tree were also self-compatible and *Dendrobium* species evolved 36.7 million years ago (Mya) [31]. Therefore, we suggest that SI in *Dendrobium* also evolved recently, and might be *Dendrobium*- or Orchidaceae-specific.

## 4. Materials and Methods

### 4.1. Plant Material

All of the species were grown under natural conditions in the Orchid Conservation and Research Centre of Shenzhen, south of China, except *D. longicornu* which was planted in Malipo, Yunnan Province. We selected 26 species using the following standards: (1) healthy plants with enough flowers (at least 10 plants); and (2) selected species needed to be in different phylogenetic positions in *Dendrobium* and cover all the *Dendrobium* clades.

### 4.2. Artificial Pollination Experiment

Flowering time was calculated based on the start time of blooming and abscission. At least five flowers of the same species were used.

Flowers were selected after 2 and 3 days of blooming to perform hand-pollination, and pollination was performed at 9:00–11:00 a.m. and 3:00–5:00 p.m. The lip was broken off by tweezers, the anther cap was removed, and then we moved the pollinium to the stigma cavity. We changed to new tweezers when completing every single pollination. All the tools were sterilized. Finally, we recorded the time of capsule formation and abscission.

### 4.3. Observation of Pollen Tube Growth

Styles to be observed were fixed in 8:1:1 of 80% ethanol, glacial acetic acid, and formalin solution at 4 °C overnight. Then, they were rinsed in 70% alcohol, softened in a strong (8 N) sodium hydroxide solution for 3 h and cleared in distilled water. Next, staining was accomplished in a 0.1% solution of water-soluble aniline blue dye in 0.1 N K_3_PO_4_ overnight. Sample staining was observed using fluorescence microscopy (Leica DM5000 B, Leica, Germany).

### 4.4. The Distribution Pattern of Self-Compatibility in the Dendrobium Phylogenetic Tree and Ancestral Status Reconstruction

Mesquite 3.4.0 [32] software was used for analyses with the maximum parsimony method. The Asian *Dendrobium* clade phylogenetic tree was constructed using the Bayesian inference method with plastid markers (*matK*, *rbcL*, *trnH*-*psbA* spacer, *trnL* intron, *atpI*-*atpH*, and *trnS*-*trnG*) and the internal transcribed spacers from the nuclear ribosomal DNA (ITS). We divided the traits into self-incompatible and self-compatible.

## 5. Conclusions

We investigated the flowering times and rates of capsule setting after self- and cross-pollination of 26 *Dendrobium* species over 2 years. We observed pollen tube growth in nine out of the 26 species and reconstructed their ancestral status in *Dendrobium*. Finally, we concluded that there was unexpectedly high diversity, not only in the rate of capsule setting but also in the pollen tube growth phenotypes. Based on the distribution pattern, we suggest that the SI type of *D. chrysanthum* was the main SI type in *Dendrobium* species. In addition, we suggest that SI in *Dendrobium* has undergone many independent evolutionary processes and evolved recently, unlike S-RNase-based GSI, which evolved over 120 Mya (resulting in the split of Asteridae and Rosidae). This study offers new insights into the SI diversity in *Dendrobium* species, which is helpful for understanding the SI determinants and provides a new perspective for interpreting the extraordinarily diverse orchids.

## Figures and Tables

**Table 1:** Pollination results of experimental *Dendrobium* species.

Species	Flowering Time (day)	Self-Pollination						Cross-Pollination					
		2016			2015			2016			2015		
		NPF	NCS	Result	NPF	NCS	Result	NPF	NCS	Result	NPF	NCS	Result
*Dendrobium aduncum*	8	75	1	1.3	50	2	4	22	3	13.6	47	8	17
*Dendrobium aphyllum*	10	58	0	0	86	0	0	56	30	53.5	84	63	75
*Dendrobium cariniferum*	21	50	0	0	46	1	2	61	20	32.7	50	23	46
*Dendrobium catenatum*	8	39	4	10	45	5	11	30	12	40	47	11	23
*Dendrobium chrysanthum*	10	100	3	3	100	0	0	100	100	100	100	100	100
*Dendrobium chrysotoxum*	9	75	68	90	100	87	87	52	52	100	89	86	97
*Dendrobium crystallinum*	7	13	11	84.6	16	15	94	20	18	90	25	22	88
*Dendrobium denneanum*	8	10	10	100	30	23	76.6	56	45	80	50	46	92
*Dendrobium densiflorum*	7	65	0	0	46	0	0	58	58	100	60	60	100
*Dendrobium devonianum*	7	100	0	0	100	0	0	100	0	0	100	0	0
*Dendrobium farmeri*	6	57	0	0	80	0	0	60	60	100	86	80	93
*Dendrobium fimbriatum*	8	60	6	10	50	5	10	56	50	89	68	62	91
*Dendrobium gratiosissimum*	9	62	0	0	52	1	2	62	62	100	68	65	96
*Dendrobium hainanense*	7	10	4	40	43	15	35	10	6	60	41	17	41
*Dendrobium hancockii*	7	24	0	0	25	0	0	8	8	100	27	27	100
*Dendrobium harveyanum*	9	65	13	20	50	12	24	45	45	100	56	50	89
*Dendrobium hercoglossum*	7	51	29	57	100	30	30	58	9	15.5	101	36	36
*Dendrobium jenkinsii*	8	66	0	0	78	6	0	59	59	100	39	27	69
*Dendrobium linawianum*	14	30	30	100	50	48	96	46	46	100	30	22	73
*Dendrobium lindleyi*	8	100	5	5	100	0	0	100	100	100	100	100	100
*Dendrobium longicornu*	11	100	0	0	100	0	0	100	100	100	100	100	100
*Dendrobium moniliforme*	9	44	0	0	46	0	0	44	18	41	47	16	34
*Dendrobium polyanthum*	10	100	0	0	87	0	0	100	98	98	80	76	95
*Dendrobium stuposum*	6	11	0	0	9	0	0	8	3	37.5	10	5	50
*Dendrobium thyrsiflorum*	9	147	6	4	100	12	12	74	74	100	71	69	97
*Dendrobium unicum*	12	28	6	21.4	52	0	0	22	0	0	48	29	60

Result = Number of capsules set (NCS)/number of flowers pollinated (NPF).
